# Supplementary material for: Effects of Exposure to Indoor Fine Particulate Matter on Atopic Dermatitis in Children
Source: Int J Environ Res Public Health. 2021 Nov 1;18(21):11509. doi: 10.3390/ijerph182111509 (PMC8583533; doi:10.3390/ijerph182111509)
Supplement: Supplementary file 1 [file ijerph-18-11509-s001.zip › ijerph-1422892-supplementary.pdf]

Supplemental materials

Supplementary Table S1. Summary of indoor and outdoor environments*

		**Total**
**Indoor**	**Temperature** (˚C)	25.5 ± 2.6
	**RH** (%)	47.2 ± 12.5
	**Formaldehyde** (ppb)	13.6 ± 20.1
**Outdoor**	**Temperature** (˚C)	14.0 ± 9.5
	**RH** (%)	67.9 ± 15.8
	**$PM_{2.5}$** ($\mu g/m^3$)	20.0 ± 11.8

*Data are expressed as mean ± standard deviation; $PM_{2.5}$, particulate matter with an aerodiameter less than 2.5 μm; RH, relative humidity

Supplementary Table S2**.** Percent changes of atopic dermatitis symptoms caused by $PM_{2.5}$ exposure

**Classification**	**Subgroup**	**% change (95% confidence interval) †**
**All**		0.86 (-2.14, 3.96)
**Season**	Spring	16.52 (6.48, 27.51)*
	Summer	-3.70 (-13.34, 7.02)
	Fall	-7.13 (-14.25, 0.58)
	Winter	12.60 (4.32, 21.53)*
**Indoor RH**	<40%	1.65 (-3.86, 7.47)
	40-60%	-0.18 (-4.49, 4.32)
	≥60%	6.99 (-5.2, 20.76)
**Indoor temperature**	<25.5°C	6.70 (2.33, 11.25)*
	≥25.5°C	-0.22 (-5.57, 5.44)
**Air purifier**	(-)	14.94 (3.46, 27.70)*
	(+)	-0.20 (-3.34, 3.03)

†% change in AD symptoms per 10 $\mu g/m^3$ of $PM_{2.5}$ exposure; *statistically significant with 95% confidence interval; RH, relative humidity.

Supplementary Table S3**.** Percent changes of atopic dermatitis symptoms caused by $PM_{2.5}$ exposure

**Classification**	**Subgroup**	**% change (95% confidence interval) †**
**All**		0.86 (-2.14, 3.96)
**Sex**	Boys	4.91 (1.36, 8.59)*
	Girls	-12.36 (-18.05, -6.28)*
**Age**	<6 yrs	-2.71 (-6.08, 0.78)
	≥6 yrs	11.96 (5.28, 19.05)*
**Family history of allergic diseases**	(-)	-2.16 (-6.68, 2.57)
	(+)	3.58 (-0.48, 7.82)
**Inhalant allergen sensitization**	(-)	-7.23 (-11.70, -2.54)
	(+)	6.96 (1.88, 12.31)*
**SCORAD at enrollment**	<30.7 (median)	1.73 (-2.33, 5.96)
	30.7-40.9	-0.80 (-7.30, 6.16)
	≥40.9 (Q3)	4.16 (-2.45, 11.23)
**Inhalant allergen sensitization(+) PLUS SCORAD**	<30.7 (median)	4.37 (-1.63, 10.74)
	≥30.7 (median)	15.68 (4.50, 28.06)*
**Air purifier**	(-)	14.94 (3.46, 27.70)*
	(+)	-0.20 (-3.34, 3.03)

†% change in AD symptoms per 10 $\mu g/m^3$ of $PM_{2.5}$ exposure; *statistically significant with 95% confidence interval; SCORAD, SCORing Atopic Dermatitis.

Supplementary Table S4. Indoor and outdoor $PM_{2.5}$ levels by season during study period

	**Indoor***	**Outdoor***	**Correlation coefficient**	***P* value***
**Spring**	28.0 ± 24.8	21.9 ± 10.4	0.22	-
**Summer**	20.8 ± 15.0	17.1 ± 9.1	0.47	<.0001
**Fall**	22.9 ± 19.3	10.0 ± 16.0	0.42	<.0001
**Winter**	47.1 ± 29.6	27.8 ± 14.6	0.32	<.0001

* Mean ± SD

Supplementary Table S5. Characteristics of AD patients and indoor environments by age group

	< 6 yrs (47 patients)	≥ 6 yrs (17 patients)	P-value
SCORAD at enrollment	33.6 ± 14.1	34.3 ± 12.8	0.6147
Inhalant allergen sensitization	41.9%	71.4%	0.2952
BMI	16.7 ± 2.0	18.3 ± 3.2	0.0651
$PM_{2.5}$ (μg/m3)	28.6 ± 24.4	29.1 ± 23.9	0.4362
Indoor temperature (°C)	25.5 ± 2.7	25.3 ± 2.3	< .0001
Indoor RH (%)	47.3 ± 1.9	46.9 ± 14.0	0.2148

SCORAD, SCORing Atopic Dermatitis; BMI, body mass index

Supplementary Fig. S1. Comparison of the $PM_{2.5}$ concentrations measured by SSP100 with those by Grimm PAS (PM2.5.prd indicates calibrated SSP100-$PM_{2.5}$ and PM2.5.grm Grimm PAS- $PM_{2.5}$)

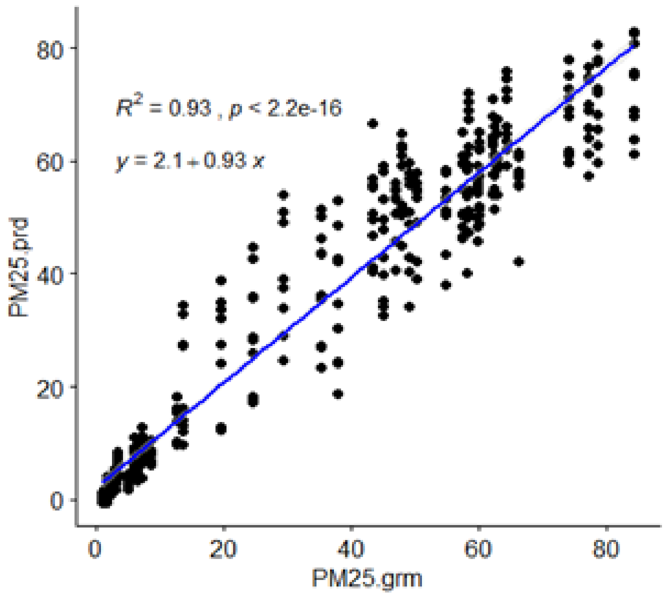

To assess indoor exposure to $PM_{2.5}$, a laser-based light scattering sensor, SSP100 (SENKO, Osan, Korea), was used. To calibrate the SSP100-$PM_{2.5}$, the assessment of the $PM_{2.5}$ sensor was performed in a room chamber with the size of 18 m^3 by comparing with Grimm portable aerosol spectrometer (PAS) 1.108 (Grimm Aerosol Technik GmbH & Co. KG, Ainring, Germany) which is widely used to assess the exposure to $PM_{2.5}$. Ten SSP100-$PM_{2.5}$ sensors and a Grimm PAS were placed into the room chamber and a certain amount of aerosol was generated by an aerosol generator, PARTICLE GENERATOR 8026 (TSI, Shoreview, USA). After conducting the test for continuous two days, we collected 10-minute data from the 10 sensors and averaged them to an hour $PM_{2.5}$. The hourly SSP100-$PM_{2.5}$ data were matched with the Grimm PAS-$PM_{2.5}$ and were stratified into four groups by concentration, 0~10, 10~20, 30~40, and > 40 $\mu g/m^3$ of $PM_{2.5}$. The regression parameters between the SSP100-$PM_{2.5}$ and

Grimm PAS-$PM_{2.5}$ were obtained based on linear regression models for each group. Fig. S1 shows that the measured values by SSP100-$PM_{2.5}$ were very consistent with the reading value of Grimm PAS-$PM_{2.5}$. The coefficient of determination (R^2) between two measured values was validated higher than 0.93. We then calibrated all the real-time SSP100-$PM_{2.5}$ measured at households applying the four parameters for $PM_{2.5}$ level.

Supplementary Fig. S2. Correlations between atopic dermatitis (AD) symptoms and environmental variables.

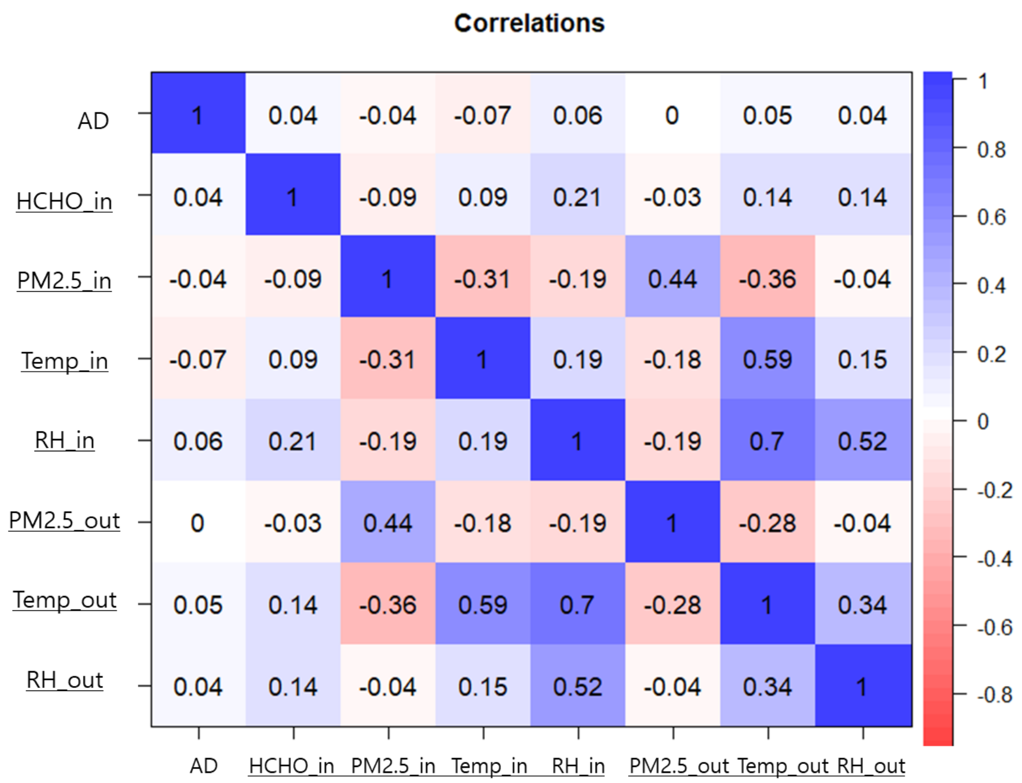

(AD: atopic dermatitis (AD) symptoms; HCHO_in: indoor formaldehyde; PM2.5_in: indoor $PM_{2.5}$; Temp_in: indoor temperature; RH_in: indoor relative humidity; PM2.5_out: outdoor $PM_{2.5}$; Temp_out: outdoor temperature; RH_out: outdoor relative humidity)
